# Little evidence of adaptation potential to ocean acidification in sea urchins living in “Future Ocean” conditions at a CO_2_ vent

**DOI:** 10.1002/ece3.5563

**Published:** 2019-08-18

**Authors:** Sven Uthicke, Nandan P. Deshpande, Michelle Liddy, Frances Patel, Miles Lamare, Marc R. Wilkins

**Affiliations:** ^1^ Australian Institute of Marine Science Townsville Qld Australia; ^2^ Systems Biology Initiative School of Biotechnology and Biomolecular Sciences University of New South Wales Sydney NSW Australia; ^3^ Department of Marine Science University of Otago Dunedin New Zealand

**Keywords:** calcifying invertebrates, carbon dioxide vents, ocean acidification, population genomics

## Abstract

Ocean acidification (OA) can be detrimental to calcifying marine organisms, with stunting of invertebrate larval development one of the most consistent responses. Effects are usually measured by short‐term, within‐generation exposure, an approach that does not consider the potential for adaptation. We examined the genetic response to OA of larvae of the tropical sea urchin *Echinometra* sp. C. raised on coral reefs that were either influenced by CO_2_ vents (pH ~ 7.9, future OA condition) or nonvent control reefs (pH 8.2). We assembled a high quality de novo transcriptome of *Echinometra* embryos (8 hr) and pluteus larvae (48 hr) and identified 68,056 SNPs. We tested for outlier SNPs and functional enrichment in embryos and larvae raised from adults from the control or vent sites. Generally, highest *F*_ST_ values in embryos were observed between sites (intrinsic adaptation, most representative of the gene pool in the spawned populations). This comparison also had the highest number of outlier loci (40). In the other comparisons, classical adaptation (comparing larvae with adults from the control transplanted to either the control or vent conditions) and reverse adaptation (larvae from the vent site returned to the vent or explanted at the control), we only observed modest numbers of outlier SNPs (6–19) and only enrichment in two functional pathways. Most of the outliers detected were silent substitutions without adaptive potential. We conclude that there is little evidence of realized adaptation potential during early development, while some potential (albeit relatively low) exists in the intrinsic gene pool after more than one generation of exposure.

## 1 INTRODUCTION

Increased sea surface temperatures (SST) and ocean acidification (OA), as a result of rising Pco_2_ concentrations, are driving changes in marine ecosystems. Average tropical SST have increased 0.4°C since the 1950s (Lough, 2012) and, compared to pre‐industrial conditions, are predicted to be 1.1–4.8°C higher by 2100 (Collins et al., 2013) with even greater increases regionally (Hobday & Lough, 2011). Such warming is leading to severe ecosystem impacts such as widespread coral bleaching (Hughes et al., 2017). At the same time, CO_2_ absorbed into the marine environment increases Pco_2_ and has reduced seawater pH by 0.11 units over 250 years. By the end of the century, Pco_2_ is forecast to increase to ~750–900 ppm and decrease ocean pH by 0.2–0.4 units (Meinshausen et al., 2011; Orr et al., 2005). Many marine organisms that rely on calcification for growth or protection may be severely affected (Doney et al., 2012) by changes in carbonate chemistry. These groups include molluscs, foraminifera, echinoderms, and corals, and their life‐history processes from fertilization success to larval development and settlement and adult growth have been reported to be deleteriously affected by OA (Anthony, Kline, Diaz‐Pulido, Dove, & Hoegh‐Guldberg, 2008; Byrne, Lamare, Winter, Dworjanyn, & Uthicke, 2013; Kroeker, Kordas, Crim, & Singh, 2010; Uthicke, Momigliano, & Fabricius, 2013; Uthicke, Pecorino, et al., 2013). There is growing interest in the role of adaptation in determining the outcomes of OA for marine taxa (Kelly & Hofmann, 2013), and particularly the mechanisms that underlay adaptation such as selection or phenotypic plasticity (Donelson, Salinas, Munday, & Shama, 2018). This includes understanding the potential for nongenetic and genetic inheritance of traits that impart greater resilience to elevated Pco_2_, such as epigenetic patterning (Hofmann, 2017; Liew et al., 2018) and allelic shifts (Pespeni, Chan, Menge, & Palumbi, 2013; Pespeni, Sanford, et al., 2013). Conversely, carryover effects may drive transgenerational responses to OA, with both positive (Hettinger et al., 2013, 2012) and negative (Donelson, Wong, Booth, & Munday, 2016; Dupont, Dorey, Stumpp, Melzner, & Thorndyke, 2013) carryover effects reported in the offspring of parents acclimated to reduced pH.

Most tests of OA on marine invertebrates, however, have been conducted on individuals not acclimatized to these altered experimental conditions, and understanding the potential for adaptation of organisms is a major knowledge gap (Kelly & Hofmann, 2013; Riebesell & Gattuso, 2015). In fact, there are a number of studies that point to the potential for adaptation to reduced pH across a range of invertebrate taxa, for example, in bivalves (Zhao et al., 2018), crustaceans (Thor & Dupont, 2015) and echinoderms (Kelly, Padilla‐Gamiño, & Hofmann, 2013). Similarly, experimentation on taxa living in naturally occurring CO_2_ vent communities can increase our understanding of the acclimation potential of species both within (Uthicke et al., 2016) and across generations (Lamare, Liddy, & Uthicke, 2016).

Next‐generation sequencing of individuals or populations allows novel insights into population structures and the potential for lineages to adapt to potential environmental stressors over the longer term. Tools for the analysis of RNA or DNA samples from pooled populations makes these tasks more time and cost effective (Kofler, Pandey, & Schlötterer, 2011; Schlötterer, Tobler, Kofler, & Nolte, 2014). In organisms with short generation times (e.g., *Drosophila*, Tobler et al., 2013), “Evolve and Resequencing” studies can be conducted, where changes in allele frequencies for a large number of single nucleotide polymorphisms (SNPs) and outlier analysis can identify which genes are under adaptive pressure, and how many generations would be required for adaptation (Kofler & Schlötterer, 2013). Similarly, physiological responses can be measured after exposing many generations to altered OA conditions (Sunday et al., 2014). In the marine realm, this can be achieved for short‐generation taxa such as coccolithophorids, where growth under different climate change conditions was observed over 460 generations (Schluter et al., 2014).

In marine studies to date, pool‐seq approaches have been mainly applied in fishes. For instance, stickleback (*Gasterosteus aculeatus*) populations in the Baltic Sea exhibited genetic differentiation which was consistent with local adaptation to temperature and salinity gradients (Guo, DeFaveri, Sotelo, Nair, & Merilä, 2015). A similar approach has been applied to Herring populations (*Clupea harengus*) which exhibited many outlier loci with apparent adaptation to local environmental conditions (Guo, Li, & Merilä, 2016). RNA‐seq approaches can also be applied to gene expression studies, for example, to investigate changes in heat stress tolerance in Copepods (Schoville, Barreto, Moy, Wolff, & Burton, 2012).

For populations of the sea urchin *Strongylocentrotus purpuratus*, Pespeni, Garfield, Manier, and Palumbi (2012) used genome‐wide restriction site tiling analysis and gene expression of pooled populations to identify loci under selection in individual populations. The study identified significant allelic changes in more than 40 functional protein classes and the authors concluded that this sea urchin has a high capacity for fast adaptation to OA conditions and that “…standing genetic variation could be a reservoir of resilience to climate change.” Follow‐up studies also used a pool‐seq approach for SNP‐based population genomics (Pespeni, Chan, et al., 2013; Pespeni, Sanford, et al., 2013) and differential expression studies (Evans, Chan, Menge, & Hofmann, 2013; Evans, Pespeni, Hofmann, Palumbi, & Sanford, 2017) to investigate the potential of that species for adaptation to ocean acidification along environmental pH gradients and in controlled laboratory experiments.

Sea urchins (Echinodermata: Echinoidea) are considered “keystone species” in many marine ecosystems and have important ecological functions as habitat modifiers, especially through macroalgal grazing (Lessios, 2001). For example, die‐off of a Caribbean species has resulted in overgrowth of coral areas with algae and an ecosystem phase‐shift (Hughes, 1994). In contrast, increased urchin abundances resulting from predator overfishing can lead to increased bioerosion and to reduced coral reef recovery after disturbances (Carreiro‐Silva & McClanahan, 2001). Increased urchin abundances can also cause “deforestation” of temperate kelp forest, ultimately resulting in urchin barrens (Steneck et al., 2002).

Experimentation on the effects of ocean acidification on sea urchins have been relatively extensive, with the response in planktonic larvae more distinct among species and ecosystems compared with those seen in adults (Byrne et al., 2013). The tropical coral inhabiting sea urchin genus *Echinometra* is relatively well‐studied. The effects of OA on adult *Echinometra* growth depend on species and experimental setup. A strong decline in growth with increasing Pco_2_ was reported for a Caribbean species (Courtney, Westfield, & Ries, 2013), and *Echinometra* species often demonstrated a reduction in growth of the larval skeleton with increased Pco_2_ and more asymmetric larvae (Kurihara & Shirayama, 2004; Lamare et al., 2016; Uthicke, Soars, Foo, & Byrne, 2013). By contrast, the effects on several Indo‐Pacific species were only subtle (Uthicke, Liddy, Nguyen, & Byrne, 2014; Uthicke, Soars, et al., 2013) or were not detected (Hazan, Wangensteen, & Fine, 2014; Moulin et al., 2015; Moulin, Grosjean, Leblud, Batigny, & Dubois, 2014).

The effects of OA on developmental stages of marine invertebrates, such as sea urchins, are usually investigated in relatively short‐term (weeks to months) controlled aquarium experiments. A recent alternative to laboratory experiments, and which allows long‐term exposures and observations, is to study organisms or ecosystems at CO_2_ vents in the field. This also considers secondary ecological effects of OA (e.g., increased food availability due to enhanced algal or seagrass growth). Vents used for these studies now include Mediterranean rocky shores (Hall‐Spencer et al., 2008), White Island New Zealand (Nagelkerken, Russell, Gillanders, & Connell, 2015), and Mariana Islands and Papua New Guinea (PNG) for coral reef environments (Enochs et al., 2015; Fabricius et al., 2011). We previously investigated sea urchin adult growth (Uthicke et al., 2016) and larval development (Lamare et al., 2016) in *Echinometra* sp. C at the well‐studied vent sites on Papua New Guinean coral reefs. Research in CO_2_ vents can be exploited to explore the phenotypic and genotypic responses in *Echinometra* offspring of acclimated animals.

These vents release nearly 100% pure CO_2_ gas into a coral reef systems and are seen as ideal “natural laboratories” to predict future responses to OA conditions (Fabricius et al., 2011). Surprisingly, for adult urchins we found increased growth rates at the vent sites. This was despite a low pH that is similar to that expected at the end of this century (Uthicke et al., 2016). By contrast, in situ* Echinometra* larval growth studies in these sites showed that exposure to future OA conditions can lead to stunted and abnormal growth (Lamare et al., 2016). This is in agreement with previous laboratory studies on the same genus (Uthicke, Soars, et al., 2013) and on a wide variety of sea urchins from tropical to arctic regions (Byrne et al., 2013).

**Figure 1:**
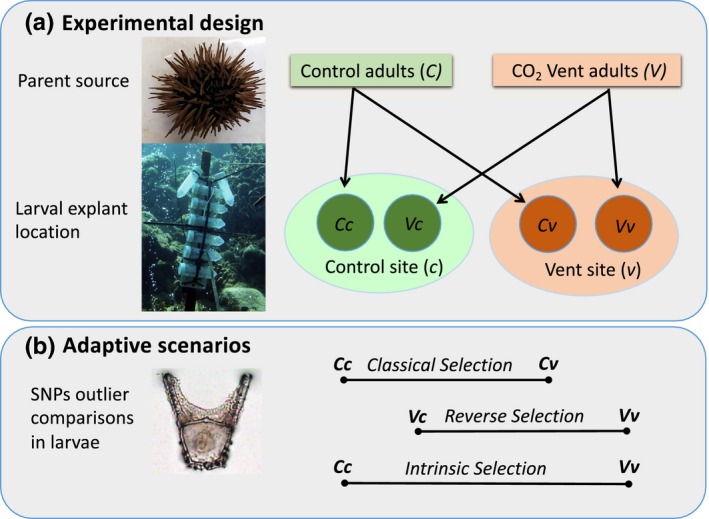
(a) Design of field experiments, with adult *Echinometra* sp. C collected from either Control (C) or Vent (V) sites, spawned, and their offspring explanted to both control locations (c) or vent locations (v). This gave rise to larvae with four histories (i.e., Cc = larvae from control adults explanted at control sites; Cv = larvae from control adults explanted at vent sites; Vc = larvae from vent adults explanted at control sites; Vv = larvae from vent adults explanted at vent sites). (b) Three potential Adaptive scenarios to changes in seawater pH for *Echinometra* sp. C larvae can be explored by detecting outlier SNPs between larvae of different histories. Classical adaptation (Cc vs. Cv) would indicate a potential for transgenerational adaptation at the genes carrying these outliers; Reverse adaptation (Vc vs. Vv) would indicate the potential for adaptation from ocean acidification to present‐day conditions, and Intrinsic adaptation (Cc vs. Vv, representative of standing genetic variation) represent long‐term (one full generation) intrinsic adaptation of the population at the vents. Classical adaptation and Reverse adaptation were tested after 8 and 48 hr of exposure. Intrinsic adaptation was only tested for 8 h larvae; SNPs in these larvae represent the closest approximation to allele frequencies in the parent populations from the control and the vent sites

Here, we exposed larvae in situ to conditions resembling OA and to present‐day seawater (control) conditions at a CO_2_ vent system in Papua New Guinea. We present a de novo assembled transcriptome of early embryos (8 hr) and 48 hr old pluteus larvae of *Echinometra* sp. C. Subsequently, we investigated the potential of adaptation of these urchins at the vent sites using a population genomic approach. Using >60,000 SNPs and outlier analysis, we investigated genes and functional groups under apparent selection. We compared larvae derived from adult urchins from either a control or vent location that were cross transplanted to the reciprocal site with those exposed at their parent's origin (Figure 1). This design should allow us to distinguish outlier SNPs in genes providing potential for adaptation to OA during short incubations, from those already differentiated in the two source populations.

## 2 MATERIALS AND METHODS

### 2.1 Species and study sites

Experiments were conducted on *Echinometra* sp. C sensu McCartney, Keller, and Lessios (2000) as evidence by cytochrome oxidase subunit 1 (CO1) sequencing (Uthicke et al., 2016). In situ larval experiments were conducted at two sites in Milne Bay Province, PNG (Fabricius et al., 2011). These consist of a CO_2_ vent site (9.82426°S, 150.81788°E), and an adjacent ambient control site ~500 m from the vent (9.82807°S, 150.82027°E) at Upa‐Upasina, NW Normanby Island (see Figure 1 in Lamare et al., 2016). The pH and Pco_2_ at the control site (pH_T_: 7.92–7.92, Pco_2_: 410–450 ppm) corresponds to present‐day conditions, whereas average pH and Pco_2_ values at the vent site (pH_T_: 7.57–7.64_,_, Pco_2_: 800–1,200 ppm) corresponds to future ocean acidification scenarios for the year ≈2100 under the RCP8.5 scenario (Meinshausen et al., 2011). There was no difference in water temperature during the deployments; average values for the control were 28.51, and for the vent 28.44°C (Lamare et al., 2016), while previous studies did not detect a long‐term difference in temperature between the control and vent site (Uthicke, Momigliano, et al., 2013). Details on carbon chemistry during the experiments are given in Lamare et al. (2016), where we also described the experimental setup and developmental responses of the larvae after 24 and 48 hr. We conducted four experimental runs (V = vent sites, C = control sites), with two runs using adults from the vent location (V1: 15‐17/11/2014; V2: 17‐19/11/2014), and two runs with adults from the control location (C1: 16‐18/11/2014; C2: 18‐10/11/2014) (Figure 1). For each experimental run, adults (~25) were collected at either the control or vent site and spawned by injecting 0.5–1.0 ml of 0.5 M KCl. This procedure yielded 10 females and 8–9 males per experimental run. Oocytes were cleaned and pooled, keeping the number of eggs per female approximately the same. Similarly, 1 µl of dry sperm from each male was mixed and the total activated in 10 ml filtered seawater and oocytes subsequently fertilized at a final concentration of 10^5^ sperm/ml. Following fertilization, embryos (700–900 individuals per replicate) for each experimental run were out‐transplanted in replicated (*N* = 6) meshed 50 ml cages either to their original location or to a reciprocal location (i.e., embryos from control populations to vents sites, and vice versa). The larvae were then left to develop in situ for 48 hr, recollected, concentrated and immediately fixed in 8 subsamples of 2 ml RNAlater, stored at 4°C for 24 hr, then kept at −20°C until required. The 48h larvae were in the 2–4 arm pluteus stage.

To observe genetic responses in earlier life stages (i.e., up to gastrula), we prepared eight, 200 ml plastic jars with both control and vent water and added 700 to 900 embryos. These embryo cultures were incubated for 8 hr in 60 L flow‐through aquaria to keep the temperature at ambient. At the end of the incubation, embryos were concentrated and fixed in 2 ml RNAlater as outlined above.

### 2.2 RNA extraction and illumina sequencing

A subsample of each RNAlater fixed sample was counted, which indicated that all samples contained 200–500 larvae. Subsamples were combined and vacuum filtered onto a Durapore^®^ membrane filter (0.45 µm; Merck Millipore). Each filter was incubated in 1.5 ml TRIzol^®^ (Invitrogen) over night. After vortexing and centrifugation (4°C, 90 s, 10,000 *g*), 1.3 ml of the resulting supernatant was pipetted into a new tube and 260 µl chloroform added. Samples were then mixed, re‐centrifuged (4°C, 15 min, 12,000 *g*) and 600 µl of the resulting supernatant transferred into a new tube. Samples were then carefully inverted after adding 600 µl of 70% ethanol. RNA extracts were subsequently purified using an RNeasy Mini Kit (Qiagen). Purified RNA quality was checked by running all extracts on 2% agarose gels and determination of concentration and A260/A280 ratios on a Nanodrop™ 2000 (Thermo Fisher).

Libraries for sequencing were prepared using the TruSeq Stranded mRNA‐seq kit (Illumina), using 1 µg of RNA as input for the poly‐A pulldown followed by cDNA synthesis, A‐tailing, and adapter ligation. The libraries were enriched by 15 PCR cycles and 75 bp paired‐end sequencing was then done on an Illumina NextSeq 500 platform using the High Output v2 kit. The sequencing was carried out at the Ramaciotti Centre for Genomics at the University of New South Wales, Australia.

### 2.3 De novo transcriptome assembly and assessment

The SolexaQA toolkit (http://solexaqa.sourceforge.net/) was used to quality trim the raw reads. Quality trimmed reads from individual samples were assembled separately with the Trinity assembly program version r2012‐06‐08 (Grabherr et al., 2011) using default parameters. A Reference Annotation Based Transcript (RABT) assembly approach was adopted, where transcripts with acceptable orthology to the model sea urchin *S. purpuratus* proteins were retained. This restricted the population differentiation analysis to variations identified in highly annotated sea urchin‐specific transcripts. The assembled transcripts from individual assembles were then combined. CD‐HIT (Li & Godzik, 2006), a program for clustering nucleotide and protein sequences, was used to identify the representative transcripts to be included in the composite representative larval transcriptome. A Trinity module was used to identify near full length/full‐length transcripts when compared to the *S. purpuratus* protein set as reference. The BUSCO v3 tool (Waterhouse et al., 2017) was used for assessment of transcriptome completeness using the representation of core‐conserved genes from the eukaryotic datasets. The consensus transcriptome was functionally annotated using the Blast2GO tool (Conesa et al., 2005).

OrfPredictor (Min, Butler, Storms, & Tsang, 2005) was used to predict putative open reading frames from the assembled transcriptome.

### 2.4 Mapping

Quality‐filtered reads from each site were aligned to the assembled transcriptome separately using BWA 0.6.1 (Li & Durbin, 2009) with a maximum edit distance of two for a maximum insert size of 500 for a read pair, and one as the maximum number of alignment for reads paired properly. The mapping results in SAM format were converted into BAM format using SAMtools 0.1.18 (Li et al., 2009) and filtered for a minimum mapping quality of 20. Duplicate reads were removed using Picard Tools (http://broadinstitute.github.io/picard/). BAM files were then converted into “mpileup” format using SAMtools 0.1.18 with maximum of 1,000 reads at a position per BAM file.

To verify the identity of the target species, we downloaded the mitochondrial NADH dehydrogenase genes ND1 and ND2 of 16 *Echinometridae* species from the NCBI database. We then also extracted the sequence of these genes from our transcriptome assembly and used Mega10 to align sequences and to construct individual phylogenetic trees by the neighbor‐joining method, without distance corrections. We used 1,000 individual runs to test bootstrap support for individual clades.

### 2.5 Detection of selection footprints (outlier SNPs)

We followed best practice guidelines for Pool‐seq analysis (Schlötterer et al., 2014) for variant detection analysis of pooled populations. The duplicate datasets for each larval sample were combined for the SNP detection analysis. We employed the following steps when using PoPoolation2 (Kofler et al., 2011) along with specific parameters. The original sync file was subsampled to a target coverage of 50 reads. SNPs, which are possibly differentiated as a result of selection, were then identified using PoPoolation2 with a minimum minor allele count of 20 and coverage between 50 and 200 within each sample. Two methods were used for outlier detection. First, for each pairwise comparison *F*_ST_ values for individual SNPs were calculated using PoPoolation2. SNPs falling into the 99.5 percentile of the empirical distribution of each pairwise *F*_ST_ were identified as potentially differentiated loci following an empirical outlier detection approach (Akey et al., 2010; Guo et al., 2016). Fisher's exact test was used to determine whether any of the differences in allele frequencies were statistically significant. The negative log *p*‐values obtained here were used downstream to identify possible enrichments using the tool ErmineJ (Lee, Braynen, Keshav, & Pavlidis, 2005). Secondly, all the SNPs identified using PoPoolation2 were converted to genotype data; this was done by simulation in the PoPoolation2 environment. The output from this was exported in GenePop format, and the transcript‐specific files were merged into a single GenePop file and subsequently converted into BayeScan format. The BayeScan tool (Foll & Gaggiotti, 2008), which aims at identifying candidate loci under natural selection from genetic data and uses differences in allele frequencies between populations, was employed to estimate the posterior probability that a given locus is affected by selection. The following parameters were used for the BayeScan run: Prior odds of 10 were used to identify the top candidates of selected loci, with a thinning interval of 10, following 20 pilot runs of 5,000 iterations each, and a burn‐in length of 50,000. A higher level of stringency was introduced into the outlier detection process wherein only the SNPs identified as outliers by both methods were considered as truly differentiated loci. This should ensure a smaller possibility of false positives.

In a separate analysis, genes differentially expressed (DE) across specific conditions were identified using the tool DESeq2 (Love, Huber, & Anders, 2014) with FDR ≤ 0.05 as a threshold. Outlier SNP‐containing genes identified above, which were also found to be differentially expressed, were removed to avoid possible influence of gene expression on our results (Pespeni, Sanford, et al., 2013). This overlap was <10% for each comparison (Tables S1 and S2). A full analysis of the DE genes was not presented here because it was not the aim of the study and thus the sampling design chosen not ideal for differential expression analysis; however, all DE genes identified in the main comparisons are presented in Tables S3 and S4.

### 2.6 Identifying the coding potential of outlier SNPs

The tool “OrfPredictor” (Min et al., 2005) was used to identify open reading frames for the transcripts with and without the alternate alleles for the outlier SNPs. The SNPs with possible changes in their coding potential were highlighted and investigated further for their functional impact in possible adaptations across comparisons.

### 2.7 Enrichment analysis

Gene ontology annotations were used to check if the outlier SNPs identified with high allelic changes show enrichments for specific biological functions. We implemented the gene score resampling method from the tool ErmineJ (Gillis, Mistry, & Pavlidis, 2010) to test for correlation between functional enrichment and changes in allele frequency. Mean score was used for multiple SNPs encountered in a gene. Statistical significance was determined by using 10,000 permutations, and corrected *p*‐values for false discovery rate were computed by the Benjamini–Hochberg approach. In addition, we tested for enrichment in biomineralization genes as proposed by Pespeni, Sanford, et al. (2013) and Evans et al. (2013). We used a list of 348 biomineralization (BM) genes from Evans and Watson‐Wynn (2014) to identify BM gene orthologs in our larval transcriptome. We then tested if outlier SNPs identified were over‐represented in each of the five main comparisons using Fisher's exact test.

## 3 RESULTS

### 3.1 Larval development

*Echinometra* sp. C larvae analyzed here were from a subset of experiments reported by Lamare et al. (2016) that were undertaken at control and vent sites measuring morphometric changes at elevated Pco_2_ concentrations. To put the current experiment into context, we briefly summarize these previous findings here. Compared to controls, 48 hr larvae exposed at vents exhibited a subtle but significant reduction in size, and larvae had increased abnormal features, measured as arm‐length asymmetry. Larvae produced from adults acclimatized to ocean acidification conditions (i.e., comparing Cv and Vv larvae as defined here) did not show greater resilience to these future ocean conditions, suggesting no detectable acclimation to reduced pH in offspring from vent adults in terms of early development.

### 3.2 Reference Annotation Based Transcript (RABT) and de novo transcriptome assembly

A total of 61 Gb of sequence was generated from 16 libraries (8 larval samples, 2 replicates each). The transcriptome assembly tool Trinity was used to de novo assemble the individual larval samples (one assembly per sample), generating an average number of 74,874 transcripts. The Reference Annotation Based Transcript (RABT) assembly step was used to compare the individual transcripts against the reference sea urchin, *S. purpuratus* protein set (NCBI ID: NC_001453.1, 35,791 proteins). Transcripts showing alignments with an acceptable e‐cutoff of <1e^−10^ were retained as those from sea urchin. The tool CD‐HIT was then used to cluster similar sequences across the RABT assemblies and to retain single representations of individual clusters and singletons that were unique to individual samples. The final consensus assembly contained 26,602 transcripts. These were used as the reference transcriptome for all downstream analyses. The cleaned short read sequences and the transcriptome have been deposited in the European Nucleotide Archive (ENA) (https://www.ebi.ac.uk/ena) under the project ID PRJEB30637. The 26,602 assembled transcripts were mapped to 11,922 unique proteins in UniProt using BlastX. The basic statistics of the de novo assembly are tabulated in Table 1.

**Table 1:** De novo assembly statistics of the *Echinometra* sp. C larval transcriptome

Assembly parameter	
Number of transcripts	26,602
Total length of transcripts (bp)	54,067,276
Mean transcript length (bp)	2,032
Median transcript length (bp)	1,581
Minimum transcript length (bp)	501
Maximum transcript length (bp)	17,644
N50 (bp)	2,652
N90 (bp)	985
GC %	43.68

The tool BUSCO was used to explore the completeness of the transcriptome. It reported the presence of 277 (91%) Benchmarking Universal Single‐Copy Orthologs (BUSCO) out of the 303 eukaryotic orthologs used in the assessment. Out of these, 190 orthologs were found to be in single copy, 87 in multi copies, 12 as fragments, and 14 were missing. This indicates that the assembled transcriptome is of high quality.

Clustering of de novo assembled ND1 and ND2 genes against reference sequences from GenBank confirmed that the assembled ND1 and ND2 genes clustered with other “*Echinometra*” species for both genes (Figure S2) and most closely with another specimen of *Echinometra* sp. C.

### 3.3 Genome‐wide population differentiation

**Figure 2:**
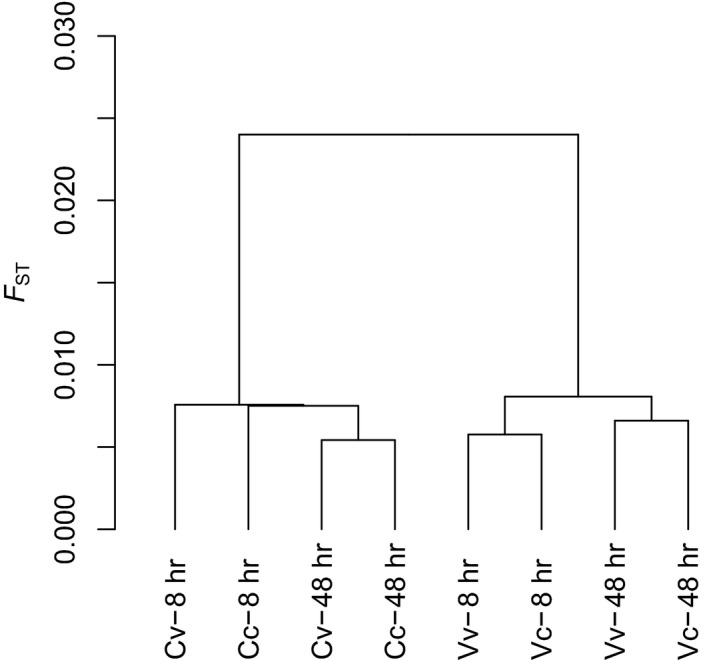
Cluster diagram of *Echinometra* sp. C larvae with parents from either control (C) or vent (V) location that have been transplanted to either the control (c) or vent (v) locations for 8 hr or 48 hr exposure before harvest. Clusters have been generated from 68,056 SNPs

PoPoolation2 identified 68,056 SNPs from all 8 one‐to‐one comparisons. *F*_ST_ values for the population comparisons ranged from 0.0054 to 0.0260 (Figure 2). Cluster analysis showed that the parental source of larvae, either control (C) or vent (V), led to the highest level of separation irrespective of larval age or transplant location (Figure 2). Within the parental location clusters, 8 and 48 hr larvae showed a slight separation, with only very subtle separation between samples deployed at their native origin or the opposite site (e.g., Cc vs. Cv).

**Figure 3:**
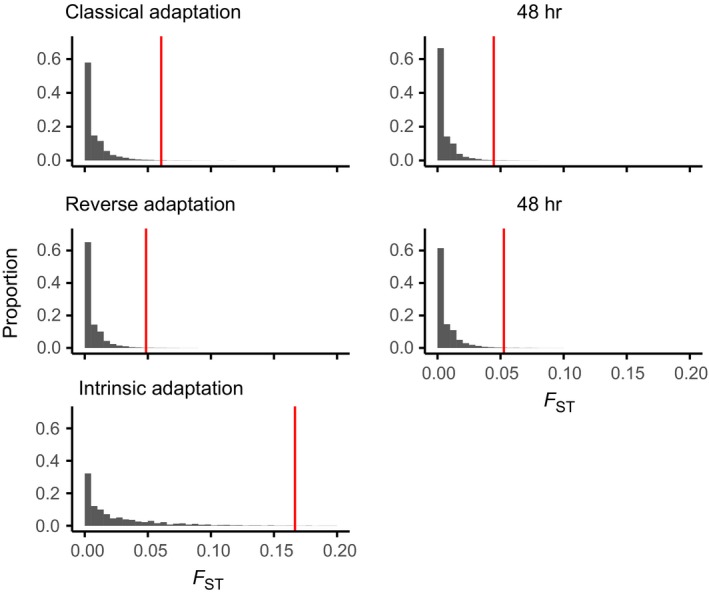
Distribution of *F*_ST_ values for classical, reverse, and intrinsic adaptation. For classical and reverse adaptation, *F*_ST_ distributions were calculated for 8 and 48 hr of exposure. Red vertical lines indicate the threshold for outliers based on the empirical outlier method. Due to the *y*‐axis scale, most outliers to the right of the vertical line are not visible. These are depicted in detail in Figure 4

We next focused on detecting outlier SNPs in the three adaptive scenarios (Figure 1b) for 8 and 48 hr, separately. First, we searched for outlier SNPs between larvae with control parental location that were back‐transplanted to control or transplanted to the vent location. This comparison transplanted to control or transplanted to the vent location (“classical adaptation,” Figure 1, Cc vs. Cv). Second, we searched for SNPs arising from the reverse of this (“reverse adaptation,” Vv vs. Vc). Third, for intrinsic adaptation or standing genetic variation, we searched for outliers between 8 hr larvae from the control parental location back‐transplanted to control and vent back‐transplanted to the vent (Cc vs. Vv). The distribution of *F*_ST_ (Figure 3) values in three adaptive scenarios confirmed the clustering in Figure 2 and average *F*_ST_ values (Table 2). *F*_ST_ distributions for intrinsic adaptation were distinctly shifted to the right as compared to classical adaptation and reverse adaptation (Figure 3). Consequently, average *F*_ST_ values and thresholds for empirical outliers are higher. For classical and reverse adaptation, the *F*_ST_ distributions matched closely for both 8 and 48 hr exposure times.

**Table 2:** Comparison of *F*_ST_ values and outlier SNPs identified for three adaptive scenarios investigated. Also shown are the results of the enrichment analysis using ErmineJ (number of enriched pathways given) and the “manual” enrichment analysis for genes involved in the biomineralization (BM) pathway (number of genes in that pathway with outlier SNPs indicated, and significance of Fisher's test)

	Comparison	Average *F*_ST_	Empirical	BayeScan	Overlap		Enrichment	BM Genes	
			SNP	SNP	SNP	Genes		*N*	*p*
Larvae 8 hr	Cc vs. Cv	0.0076	351	21	19	17	0	0	–
	Vv vs. Vc	0.0058	348	8	6	6	0	0	–
	Cc vs. Vv	0.0233	343	51	40	39	0	1	n.s.
Larvae 48 hr	Cc vs. Cv	0.0042	328	11	10	10	2	0	–
	Vv vs. Vc	0.005	351	14	11	11	0	1	n.s.

**Figure 4:**
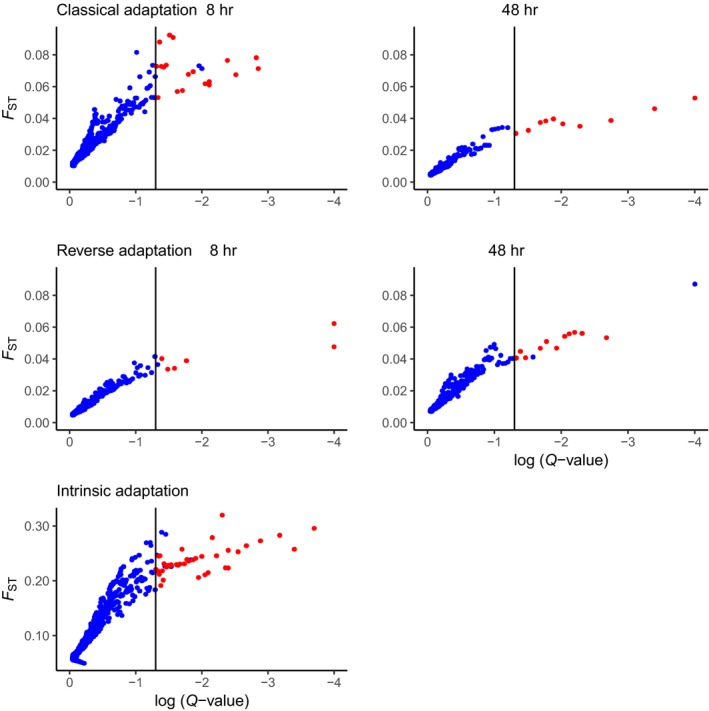
*F*_ST_ versus log *Q*‐values for classical, reverse and intrinsic adaptation. Vertical lines indicate the threshold for outliers in the BayeScan analysis (FDR = 0.05). Red dots indicate overlap outliers between the BayeScan and the empirical method

Outlier SNPs were detected in classical and reverse adaptation, for both 8 and 48 hr exposure, and for intrinsic adaptation. Using the empirical outlier approach, we detected 328–351 outlier SNPs across all larval samples (Table 2). The number of SNPs identified with BayeScan, a highly conservative approach, ranged from 8 to 73 (Figure 4) using a false discovery rate (FDR) threshold of 0.05. Most BayeScan outlier SNPs overlapped with the SNPs identified from the empirical analysis. Overlapping outlier SNPs (red dots in Figure 4) were distributed across 6–39 genes. Thus, most genes affected only had one outlier SNP (Tables 2 and 3). Most outlier SNPs and genes were detected in the intrinsic adaptation comparison based on 8 hr larvae (Table 2). All overlap outliers identified were “high” outliers, thus indicative of diversifying selection (Figure 4). We also tested whether the substitutions in the outlier SNPs would lead to amino acid changes and thus provide potential for adaptation (Table 3, details in Table S3). Only 5 out of 66 (7.6%) outliers in the 8 hr data and 2 out of 21 (9.5%) of the 48 hr outliers led to amino acid changes, the remainder being silent substitutions. Several of these genes were uncharacterized, and the remaining 5 genes are not clustered in any biochemical pathway.

**Table 3:** Outlier SNPs leading to changes in amino acids (AA) in genes. f1 and f2 are the frequencies of the minor (based on the average frequency) alleles in the respective comparisons. SNP No.: position of the SNP along the gene

	Comparison	Gene No.	SPU‐ID	Protein function	SNP No.	f1	f2	(Condon‐AA change)
Larvae 8 hr	Cc vs. Cv	HAMP01014099.1	NA	Uncharacterized protein	1555	0.22	0.64	(TTC/CTC) (F/L)
		HAMP01011896.1	013103	Uncharacterized protein	1468	0.1	0.4	(ACT/GCT) (T/A)
	Vv vs. Vc	HAMP01007373.1	021184	Atp‐binding sub‐family g member 2	1307	0.18	0.56	(GTA/GCA) (V/A)
	Cc vs. Vv	HAMP01003429.1	NA	Ribonuclease h2 subunit c	703	0	0.48	(AGT/AGG) (S/R)
		HAMP01013370.1	NA	Inactive peptidyl‐prolyl cis‐trans isomerase fkbp6	1122	0.54	0.06	(AGC/AGG) (S/R)
Larvae 48 hr	Cc vs. Cv	HAMP01003768.1	017289	Carboxy‐terminal kinesin	1043	0.14	0	(CAG/CTG) (Q/L)
		HAMP01007614.1	016156	Uncharacterized protein	747	0.42	0.14	(AAA/CAA) (K/Q)
	Vv vs. Vc	No change						

We tested for functional enrichment for individual sets of genes containing outlier SNPs for each comparison for biological pathways using ErmineJ. We did not detect any enrichment for all but one comparison in the 48h adaptation scenario (Table 2). In that comparison, we found two pathways enriched. These pathways were similar, namely “regulation of protein localization in plasma membrane” (GO:1903076) and “positive regulation of protein localization to plasma membrane” (GO:1903078). We identified 274 genes in the larval assembly as putative orthologs of genes involved in biomineralization, however, in only two of our five main comparisons did an outlier SNP occur in a biomineralization gene (Table 2). Hence, none of the comparisons had a significant enrichment of outliers in these genes.

## 4 DISCUSSION

This research tested the hypothesis that long‐term exposure of adult sea urchins to elevated Pco_2_ (intrinsic adaptation) or short‐term (8, 48 hr) larval exposure (classic adaptation) can lead to adaptation to ocean acidification conditions. We will argue that we must reject this hypothesis because: (a) compared to other studies the number of outliers detected is small; (b) most of these outliers are “silent” substitutions, and; (c) apparent outliers exhibit little enrichment for biological pathways.

Given that *Echinometra* sp. C is a nonmodel species and the only urchin genome available is from a different echinoid Family (*Strongylocentrotus*, Sodergren et al., 2006), we assembled a transcriptome for *Echinometra* sp. C from all available larvae samples. A high N50 suggested that the transcriptome was highly contiguous in its structure. The completeness of the transcriptome was demonstrated by using BUSCO, which showed that 91% of core‐conserved genes are found in the assembly. The number of unique proteins identified in our study was comparable to recently assembled sea urchin transcriptomes such as *Loxechinus albus* (11,347, Gaitán‐Espitia, Sánchez, Bruning, & Cárdenas, 2016), *Evechinus chloroticus* (11,906, Gillard, Garama, & Brown, 2014), or *Heliocidaris erythrogramma* (13,548, Wygoda, Yang, Byrne, & Wray, 2014).

For selection to act in short‐term experiments differential mortality of animals with alleles less suitable for the new environmental conditions need to occur. Although 8 and 48 hr appear short, we assumed selective pressure in this first period of embryonic and larval development to be strong, given the near universal effects of OA on growth and development of echinoid larvae in this early period (reviewed in: Byrne et al., 2013), including larvae of *Echinometra* spp. (Uthicke, Soars, et al., 2013). However, to our knowledge none of these studies investigated mortality; thus, it is possible that measured stress in these early phases does not translate into (selective) mortality. The stress could nevertheless affect fitness and capacity to grow into adulthood, and however, this was not investigated here.

By contrast, the intrinsic scenario measures selective pressure over a longer period (at least from settlement to spawning (based on growth rates in Uthicke et al. (2016) average size adults used for spawning are >3 years old), albeit not over multiple generations. This is because, given the small size of the vents, only a low proportion (if any) of larvae generated from vent adults are likely to re‐settle at the vents (Uthicke et al., 2016). Thus, in this study, selection could be acting on late larval stages during settlement and metamorphosis, and growth and development of juveniles and adults. This selection together with genetic drift may contribute to high *F*_ST_ values between locations. The *F*_ST_ value (~0.02) between the vent and control suggested a relatively high level of population differentiation given the geographic closeness of the vent and control samples. Free‐spawning echinoderms with long‐lived planktotrophic larvae usually have *F*_ST_ values indistinguishable from zero over short (<10 km) geographic differences; for example, sea cucumbers (Uthicke & Benzie, 2003), sea stars (Harrison, Pratchett, Messmer, Saenz‐Agudelo, & Berumen, 2017; Williams & Benzie, 1997), or sea urchins (Duchaud, Durieux, Coupé, Pasqualini, & Ternengo, 2019; Tourón et al., 2018). Also, SNP‐based studies generally show low population genetic structure (*F*_ST_ often < 0.002) within regions for planktotrophic echinoderms such as holothurians (Xuereb et al., 2018). Based on mitochondrial genes, intra‐regional differences between *Echinometra* sp. C can be nonsignificant (bindin gene) or range from 0.04 to 0.15 (COI, Geyer & Palumbi, 2003). An allozyme study on *Echinometra matthaei* demonstrated *F*_ST_ values around 0.01 on geographic scales from single to 1,000's of kilometers (Watts, Johnson, & Black, 1990); thus, *F*_ST_ values detected here over small distances may not be unusual but also reflect genetic patchiness.

Outliers SNPs in the intrinsic comparison were 2–4 times more common than in the other comparisons, indicating that some selection between the sites is realized. The outlier number detected in this comparison was in the same order as in an experimental study on larvae of *S. purpuratus* (~40, Pespeni, Sanford, et al., 2013) but much lower than found in adults of the same species along natural pH gradient (>300, Pespeni, Chan, et al., 2013).

A reduction of size and increased abnormality upon exposure to ocean acidification is documented in many arctic to tropical sea urchin species (Byrne et al., 2013). Morphological studies from the same experiments presented here (Lamare et al., 2016) showed that, compared to larvae exposed at controls (Cc or Vc), 48h pluteus larvae exposed at vents (Cv or Vv) exhibited a reduction in size, and larvae had increased abnormality. Offspring from adults from the vents (i.e., those acclimatized to ocean acidification; V) were equally affected by OA when larvae were back‐transplanted to the vents (Vv) compared to larvae from adults at the controls exposed at the vents (Cv). Thus, in terms of development, no evidence for selection or transgenerational phenotypic plasticity was detected in that study. Similarly, in an aquarium experiment on *Echinometra* sp. A (Uthicke, Soars, et al., 2013), larvae produced from adults pre‐acclimatized (7 weeks) to low pH were not more resilient to the effects of ocean acidification compared to larvae with parents exposed to present‐day conditions.

Evans et al. (2017) found genes in tricarboxylic acid cycle, electron transport chain, and beta oxidation pathways upregulated in adult sea urchin larvae exposed to low pH (or high Pco_2_). The authors concluded that this was an indication of ATP channeled toward maintaining acid–base homeostasis to increase tolerance to low pH, most likely at the cost of other measures of fitness. Given adults from the vent populations studied here grew faster and had a similar amount of energy channeled toward gonad production as those at controls, we saw no evidence for this in *Echinometra* sp. C adults (Uthicke et al., 2016). However, given *Echinometra *sp. larvae are generally smaller under OA and have more abnormalities (Lamare et al., 2016; Uthicke, Soars, et al., 2013) it is possible that these changes also occur, and without these, the effects of OA would be more drastic or lethal at earlier stages.

We discovered very few outliers indicative of potential adaptation. In addition, most outliers were silent substitutions that would not result in amino acid changes. The percentage of outlier SNPs leading to amino acid changes detected here (~5%–8%) is somewhat below the number identified in Pespeni, Sanford, et al. (2013) (~11%). Only one of the outlier genes causing a nonsilent substitution detected here (inactive peptidyl‐prolyl cis‐trans isomerase fkbp6, intrinsic comparison) had a homolog downregulated in *S. purpuratus* 1 day larvae under lower pH conditions (peptidyl‐prolyl cis‐trans isomerase CWC27 homolog) (Evans et al., 2017). None of the genes with nonsilent substitutions were differentially expressed on day 7 of Evan's study or were among the genes putatively under selection in other ocean acidification studies on *S. purpuratus* (Pespeni, Chan, et al., 2013; Pespeni, Sanford, et al., 2013).

Low statistical power to detect outlier loci in our study, especially for the *intrinsic* comparisons between two populations already separated by high *F*_ST_ values may be one reason for detecting less evidence for selection then Pespeni, Sanford, et al. (2013). However, we used nearly twice as many parents as Pespeni et al., and the number of larvae sequenced (>200) was well in the range recommended for “Evolve and Resequencing” studies (Schlötterer et al., 2014). Thus, at least for the “classical” and “reverse” comparison (using the same parents each, i.e., *F*_ST_ at the start of the experiment close to 0), power cannot be assumed a reason for different findings compared to Pespeni et al. Even in the “intrinsic” comparison, we detected several apparent outliers with low minor allele frequencies (<0.1, Tables S1–S4), indication that low power may also not be a major issue in that comparison.

Based on previous studies on a temperate sea urchin, it was surprising that outlier genes in our study were not enriched for the biomineralization pathway. After 7 days of exposure to OA conditions, larvae of *S. purpuratus* outliers were significantly enriched in the biomineralization pathway (Pespeni, Sanford, et al., 2013), and this pathway was also over represented in outlier SNPs along a natural pH gradient (Pespeni, Chan, et al., 2013). However, there was no enrichment of differentially expression genes in the biomineralization pathway for larvae of the former species after 1 and 7 days exposure to OA (Evans et al., 2017).

Mortality rates may depend on the duration of experiments. This may explain the lower potential for adaptation observed in the present study on *Echinomtera*, compared to *S. purpuratus* (Pespeni, Chan, et al., 2013; Pespeni, Sanford, et al., 2013). In this respect, while both studies examined SNP outliers in the same developmental stage (i.e., 4‐Armed pluteus), the timing of sampling was shorter in the present study (48 hr) compared with sampling time in (Pespeni, Chan, et al., 2013; Pespeni, Sanford, et al., 2013). It is possible, therefore, that the longer sampling duration may have allowed greater selection for genotype and hence greater differences in allele frequency seen in *S. purpuratus*. Although a previous study exhibited high (~50%) mortality in sea urchin larvae after 48 hr under OA (Gianguzza et al., 2014) it is possible that in our study, this length of time was insufficient to drive differential mortality or a resulting difference in allele frequency.

Furthermore, a potential for adaptation also requires the intrinsic presence of genetic variation leading to gene variants more suitable for altered environmental conditions. Some *S. purpuratus* populations inhabiting the west coast of North America naturally experience low pH in upwelling areas (Pespeni, Chan, et al., 2013), and possibly over many generations. Thus, standing genetic variance in that species may allow adaptation/acclimation to low pH on short time scales. Although *Echinometra* sp. on shallow coral reef flats can be exposed to low pH (<7.8) and high Pco_2_ (>1,000 ppm) at night (Uthicke et al., 2014), these short‐term exposure to future ocean conditions may not be a sufficient evolutionary force resulting in potential for rapid adaptation and selection across generation.

In conclusion, our study revealed little evidence of a potential of adaptation during the first 2 days of larval development. Although some adaptation may have occurred in the intrinsic gene pool (separate populations at vents and controls), a previous morphometric study suggested that lifetime acclimation of the parents conveyed no resilience to OA to the larvae (Lamare et al., 2016). Thus, the results from the present genetic study and the previous morphological study are congruent. The transcriptome assembly presented here will be an important resource for future adaptation studies of this keystone coral reef species.

## CONFLICT OF INTEREST

None declared.

## AUTHOR CONTRIBUTIONS

SU has designed the study, ran experiments, lead the writing and analysis. ND has performed most of the data analysis and contributed to writing. MLi ran field experiments and contributed to writing. FP conducted laboratory analysis and contributed to writing. MLa and MRW contributed significantly to the writing and data analysis writing.

## Data Availability

The cleaned short read sequences and the transcriptome have been deposited in the European Nucleotide Archive (ENA). Detailed lists of all outliers identified are in the Tables S1–S4. The cleaned short read sequences and the transcriptome have been deposited in the European Nucleotide Archive (ENA) (https://www.ebi.ac.uk/ena) under the project ID PRJEB30637. Scripts for the transcriptome assembly, mapping, and outlier detection are available on github (https://github.com/nandan75/seaurchin-population-genomics.git).
