# Supplementary material for: Little evidence of adaptation potential to ocean acidification in sea urchins living in “Future Ocean” conditions at a CO2 vent
Source: Ecol Evol. 2019 Aug 18;9(17):10004–16. doi: 10.1002/ece3.5563 (PMC6745858; doi:10.1002/ece3.5563)
Supplement: Supplementary file 2 [file ECE3-9-10004-s002.docx]

**Supplementary Figures**

**
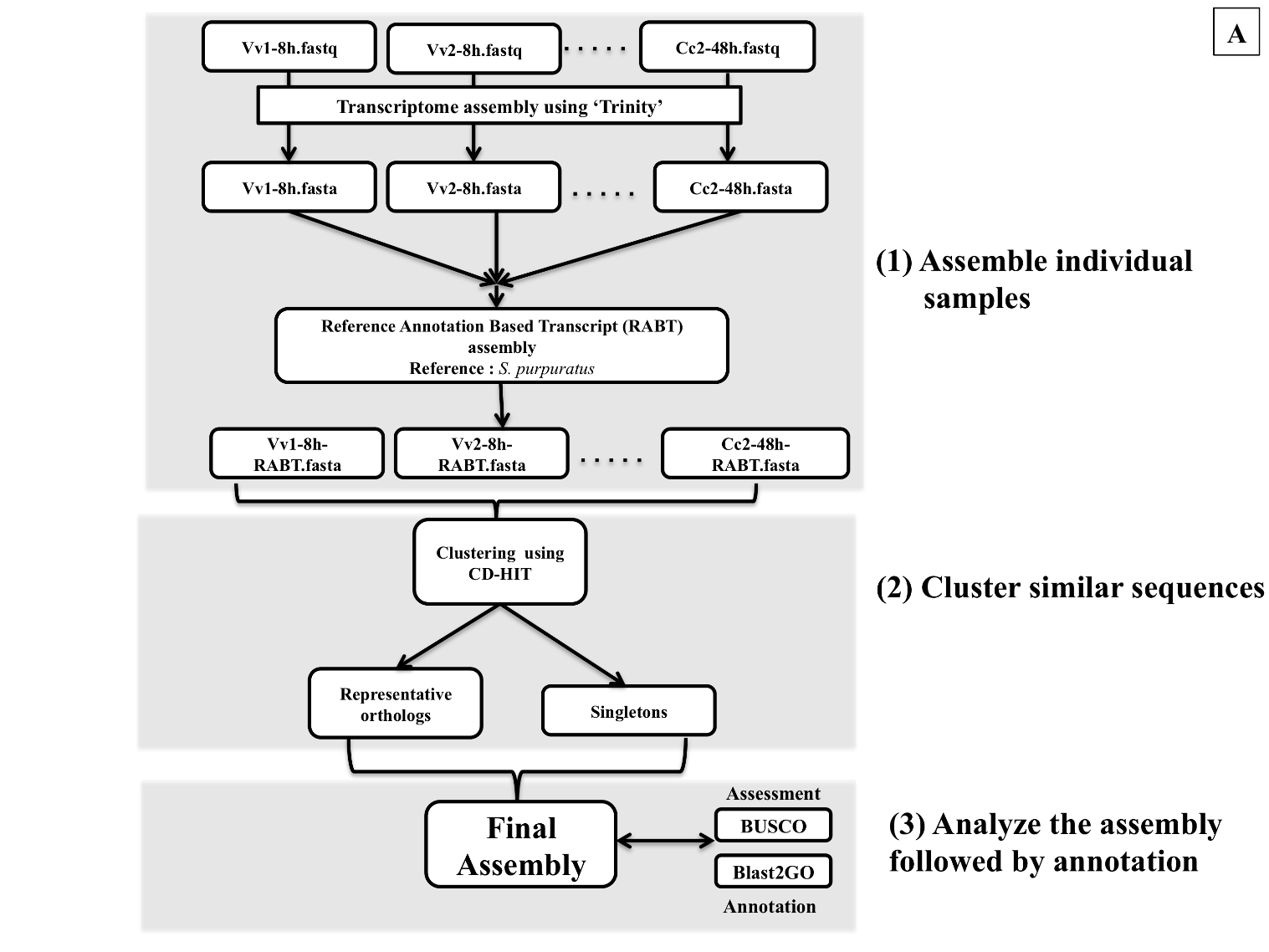
**

**
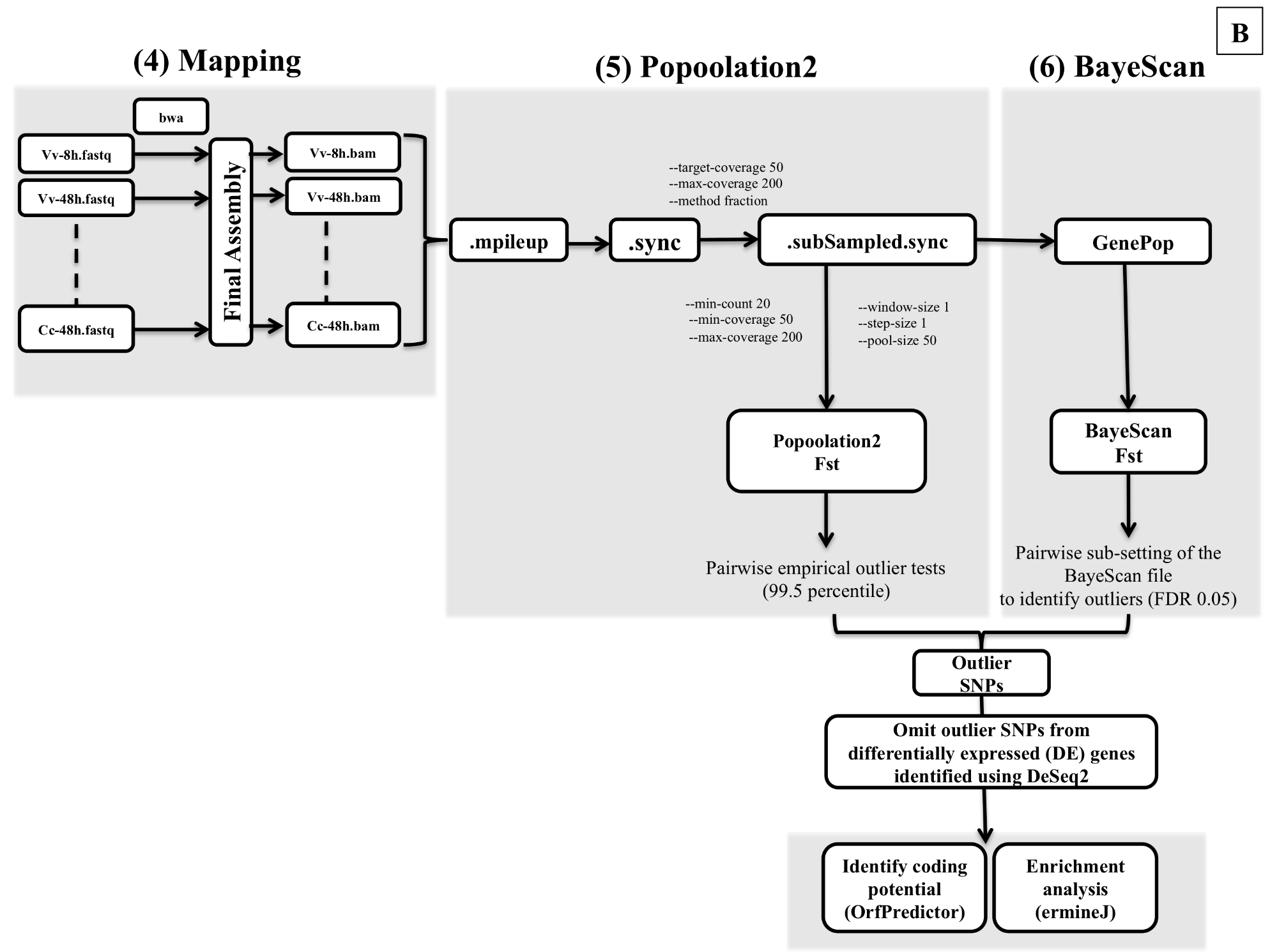
**

**Supplementary Figure 1 (A)** We adopted a Reference Annotation Based Transcript (RABT) assembly approach and generated a composite representative larval transcriptome using 16 larval samples from both control and vent locations.

The clustering tool CD-HIT was used to remove redundancies, and this was followed by assessment using the tool BUSCO and annotation using Blast2GO. **(B)** The replicate samples from a specific condition were merged before they were mapped to the transcriptome assembly using bwa. A highly stringent set of consensus outlier SNPs were identified using two different tools: Popoolation2 and BayeScan. The SNPs belonging to genes which were found to be differentially expressed using DeSeq2 were omitted. The coding potential of the outlier SNPs was identified using OrfPredictor. This was followed by enrichment analysis using ermineJ

**a) ND1**


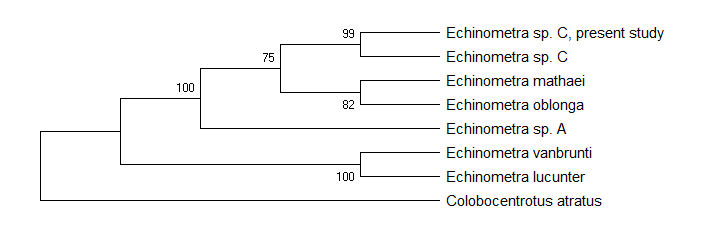


**b) ND2**


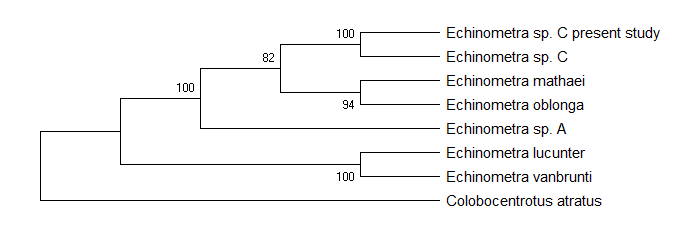


**Supplementary Figure 2** Bootstrap (N = 1000) consensus trees of ND1 (819 BP) and ND2 (1068 BP) (have tiff files, need to work on labels, Genbank Nos etc). The sequence retrieved from the present study has 99.14% (ND1) and 99.62% (ND2) similarity to sequences of *Echinometra sp. C* on Genbank, obtained by standard PCR. All sequences except from those in the present study are from Kinjo et al. (2008).

Supplementary References

Kinjo, S., Shirayama, Y., & Wada, H. (2008). Evolutionary history of larval skeletal morphology in sea urchin Echinometridae (Echinoidea: Echinodermata) as deduced from mitochondrial DNA molecular phylogeny. *Evolution & development, 10*(5), 632-641.
